# How Does Moxibustion Possibly Work?

**DOI:** 10.1155/2013/198584

**Published:** 2013-03-27

**Authors:** Jen-Hwey Chiu

**Affiliations:** ^1^Institute of Traditional Medicine, National Yang-Ming University, No. 155, Section 2, Linong Street, Beitou, Taipei 112, Taiwan; ^2^Division of General Surgery, Department of Surgery, Taipei Veterans General Hospital, Taipei 115, Taiwan; ^3^Department of Surgery, Cheng-Hsin General Hospital, Taipei 115, Taiwan

## Abstract

“Acupmoxa” is a hybrid word of “acupuncture” and “moxibustion” that more closely resembles the Chinese ideograph for this treatment. People in Western countries are more familiar with acupuncture, while moxibustion is less popular, partially due to the paucity of scientific studies. Although the evidence-based efficacy of moxibustion needs to be further clarified, the mechanisms by which moxibustion may work include temperature-related and nontemperature-related ones. Local somatothermal stimulation (LSTS), one type of moxibustion, is achieved by application of a heat source to and above the acupoint. Such mild heat stimulation of the acupoint induces little skin damage, in contrast to the burning effect of moxibustion, but does provoke mild oxidative stress in the viscera. Thus, preconditioned LSTS at the peripheral acupoints LR 14 and PC 6 of animals is able to induce visceral HSP70 expression and to protect the liver and the heart against ischemia-reperfusion injury. Nontemperature-related mechanisms include smoke, herbs, and biophysical (far infrared) stimulation. We conclude that LSTS, a remote preconditioning method, has potential clinical usefulness. However, evidence-based efficacy and safety studies involving large-scaled clinical trials are needed in order that this approach will pass muster with Western scientists.

## 1. Introduction

“Acupmoxa” is a hybrid word of “acupuncture” and “moxibustion” that more closely resembles the Chinese ideograph for this treatment. Acupuncture describes a procedure involving penetration of skin areas (acupoints) by thin metallic needles, which is followed by manipulating the needles manually. Moxibustion describes a technique that applies heat to acupoints by burning compressed powdered herbal material at the acupoints to be stimulated. Acupuncture or moxibustion, either alone or in combination, can be applied when treating patients with a wide range of diseases [1]. In 1980, the World Health Organization (WHO) recommended acupuncture as an effective treatment for forty-three health problems, including respiratory tract disorders, gastrointestinal disorders, eye disorders, and neuromuscular disorders [2]. People in the Western countries are more familiar with acupuncture; in contrast, moxibustion has remained less popular, perhaps partially due to the paucity of relevant scientific studies.

In contrast to the development of Western medicine, which can be traced back to Hippocrates via a clear and distinct route, Chinese acupmoxa theory was already fully developed by the end of the 2nd century BCE. In 1972, documents written on silk scrolls in a Ma-Wang-Dui tomb sealed in 198 BCE were discovered in China. This discovery included documents that only relate to moxibustion and do not include any references to acupuncture or acupoints. The documents refer to eleven lines of channel (meridians), which suggests that the origins of moxibustion and of meridians are earlier than those of acupuncture and acupoints [3].

## 2. The Classification and the Efficacy of Moxibustion

Classically, moxibustion is applied to patients with the use of nonmoxa or moxa sticks, and the latter can be applied either directly or indirectly [4]. Direct moxibustion is defined as application of moxa sticks onto or above the destined acupoints on the body surface, while indirect moxibustion is the application of herbs (mugwort, ginger, etc.) between moxa sticks and the acupoints. Sometimes, moxibustion can be applied over acupuncture needles [5], either with or without scarring, in order to improve efficacy [4, 6].

Based on descriptions in the ancient Chinese literature, the therapeutic effects of moxibustion are associated with treating chronic symptoms related to “deficiencies” and to the prevention of human disorders. Previous studies have demonstrated that moxibustion is effective when used to treat cervical vertigo [7], dysmenorrheal [8], chemotherapy-induced leucopenia [9], and various emergency conditions [10]. Much effort has been devoted to the studies of moxibustion using experimental tumor models, including with or without smoke [11], using different modes [12] and in combination with radiotherapy or taxol treatment [13, 14].

Since the late 20th century, it has been suggested that moxibustion increases fetal activity during the treatment period, cephalic presentation after the treatment period, and cephalic presentation at delivery [6, 15–17]. However, meta-analysis of a large number of investigations over the past two decades has failed to demonstrate that moxibustion effectively produces cephalic inversion during breech presentation [18–20] or is a useful treatment for stroke [21], hypertension [22], rheumatic conditions [23], ulcerative colitis [24], constipation [25], pain relief [26], and cancer support [27]. There is consensus that well-designed randomized controlled trials are needed in order to evaluate the safety and efficacy of moxibustion.

## 3. Possible Mechanisms of Action of Moxibustion

### 3.1. The Temperature-Related Mechanisms of Action of Moxibustion

As moxibustion is defined as a technique that applies heat to acupoints by burning herbal materials on the body surface, factors such as temperature, smoke, odor, and herbs are likely to be involved in the possible mechanisms by which moxibustion may work.

#### 3.1.1. Local Somatothermal Stimulation (LSTS)

Conventional application of moxibustion evokes multiple sensory stimulations, including temperature, pressure, pain, touch, and smoke stimuli. To avoid difficulties with respect to data interpretation when there are moxibustion-induced multisensory stimulations, Chiu et al. used temperature as the only stimulator in their series of studies (Table 1). In brief, local somatothermal stimulation (LSTS), which was compared with whole-body hyperthermia, was achieved by the application of a heat generator to and above (0.5 cm) the acupoint without any contact with the skin surface; furthermore, a fluctuating skin temperature was obtained by intermittently turning on and off the heat generator (4 minutes on and 5 minutes off for three cycles). Usually, it took 27 minutes to complete one LSTS treatment [28]. Usually, LSTS was repeatedly applied at 12-hour intervals. The fluctuation in temperature brought about by the LSTS was designed to make a temperature increase and decrease in relation to the critical point of 42°C, so that the heat-sensitive neural transmission would not be tolerated. It is important to notice that, when there is such mild heat stimulation, no skin damage such as burning injury or nerve damage can be observed.

#### 3.1.2. LSTS at Acupoints Relaxes the Sphincter of Oddi and the Anal Sphincter via the Neural Release of Nitric Oxide (NO)

Several lines of evidence support the idea that NO plays an important role in the gastrointestinal system and acts as a neurotransmitter in nonadrenergic, noncholinergic, or “nitrergic” neurons of the peripheral nervous system [36, 37]. When LSTS was applied onto and 0.5 cm above acupoint GB 24, manometry of sphincter of Oddi (SO) showed that the tonic pressure and phasic contraction pressure of this sphincter were decreased. The LSTS-induced relaxation of the SO could not be blocked by pretreatment with atropine, phentolamine, or propanolol but could be blocked by L-NAME; furthermore the blockage could be reversed by L-arginine and not by D-arginine. These findings suggest that LSTS relaxed the SO via activation of neural L-arginine/NO pathway. The effect of LSTS on SO relaxation could be observed not only in carnivorous species (the cat) and in herbivorous species (the rabbit), but also in humans [28]. In addition, LSTS at designated acupoints (BL 36 and BL 40) was also shown to relax hypertonic anal sphincters in humans [29], possibly via the nitrergic neural release of nitric oxide [30]. The responses of the SO and anal sphincters to LSTS were found to be temperature-specific (42°C) and acupoint-specific; furthermore, the neurotransmitter was nitric oxide.

#### 3.1.3. LSTS at Peripheral Acupoints Induces the Expression of Heat Shock Protein 70 (HSP70) in Corresponding Organs

It is noteworthy that the critical temperature for evoking NO-related sphincteric responses by LSTS is around 42°C, which is similar to the temperature used to induce heat shock protein (HSP) expression in many studies [38, 39]. To test the hypothesis that LSTS at peripheral acupoint without contact with the skin surface is able to induce HSP70 expression in the corresponding visceral organ, LSTS was applied onto and above acupoint LR 14 or acupoint PC 6, and the HSP70 gene expression in the liver and heart, respectively, was analyzed by Western blotting and RT-PCR. Acupoint LR 14 is innervated by the seventh intercostal nerve, and PC 6 is innervated by the median nerve; these have been used in traditional Chinese medicine for the treatment of hepatobiliary and heart disease, respectively. Lin et al. demonstrated that LSTS at the LR 14 acupoint induced HSP70 expression in the liver, but not in the heart. When analyzed by Western blotting and RT-PCR, the LSTS-induced HSP70 expression was determined to be *de novo* synthesis in the liver [31]. On the other hand, LSTS at the PC 6 acupoint induced *de novo* HSP70 expression in the heart, but not in the liver [32]. Taken together, these novel findings suggest that the LSTS-induced visceral HSP70 expression occurs in a meridian-specific manner.

#### 3.1.4. Preconditioning by LSTS Protects Organs against Ischemia-Reperfusion (I/R) Injury

Since HSP70 has been reported to enhance myocardial tolerance against I/R injury [40–42], it was reasonable to postulate that preconditioning by LSTS at peripheral acupoints ought to induce visceral HSP70 expression and protect the relevant organs from subsequent I/R injury. When animals were preconditioned with three doses of LSTS at the left PC 6 acupoint (median nerve territory) and this was followed by subsequent I/R injury to the heart, there was a significant decrease in the creatine kinase level of the heart, a significant decrease in the duration of arrhythmia, and a significant decrease in the mortality rate as well as improved mitochondrial respiratory functioning when compared to animals without prior LTST preconditioning [31]. Furthermore, when animals were preconditioned with one dose of LSTS at the right LR 14 acupoint (7th intercostal nerve territory), followed by subsequent I/R injury to the liver, there were a significant decrease in liver enzymes (ALT/AST) and a significant decrease in malonyldialdehyde (MDA) formation when compared to animals without prior LSTS treatment or to animals with three doses of LSTS treatment [39]. In addition to the above, LSTS has been used in combination with the oral administration of geranylgeranylacetone in order to bring about tolerance of I/R injury to rat livers [43].

Recently, Pan et al. used the rubber band wrapping model to induce I/R injury to the calf muscle induced via rubber band encasement; the animals underwent injury with or without preconditioning by LSTS. No significant change in neuromuscular function was found between the LSTS (−) and LSTS (+) groups on the first day after I/R injury. However, gait stride length, compound motor action potential, and the level of serum creatine phosphokinase MM isoenzyme were found to be significantly improved on the eighth day when there had been one or two doses of LSTS preconditioning compared to the situation without LSTS preconditioning. The results suggest that LSTS preconditioning protects the animals with respect to neuromuscular plasticity when there is tourniquet-induced neuromuscular injury [34].

#### 3.1.5. Effects of LSTS Occur via Somatovisceral Regulation

It is well known that viscerovisceral reflex regulation is a normal physiological response. For example, relaxation of the internal sphincter of the anus (the rectoanal reflex) is observed when rectal pressure is increased. A growing body of evidence suggests acupuncture may adjust visceral function and modulate immune response via a “Somatovisceral” mechanism [44–51]. The fact that acupuncture and related alternatives increase the concentrations of opioids and monoamines in cerebral spinal fluid and the levels of vasoactive intestinal peptide (VIP) and cholecystokinin (CCK) in serum suggests that Somatovisceral regulation occurs via neuroneural or neurohumoral pathways. It is generally accepted that local anesthetics depress completely the transmission of pain and thermal sensations, which are carried by A*δ* or C-fibers [52]. LSTS at a peripheral acupoint induces nitrergic neural release of NO in Oddi's and anal sphincters, and this is able to be completely blocked by local infiltration of an anesthetic agent at the LSTS site. Due to how embryonic development takes place, visceral pain is perceived as originating from a somatic area, a phenomenon known as “referred pain.” Previously, a role for polymodal receptors (PMRs) in this phenomenon has been suggested based on the fact that PMRs are responsive to both acupuncture and moxibustion stimuli and that thermal sensitivity is essential to moxibustion therapy [53]. These findings are in agreement with the fact that the effects of LSTS are mediated by Somatovisceral regulation via heat-sensitive sensory afferent and NANC motor neurons.

#### 3.1.6. Differences between Acupuncture and LSTS

Previous investigations have demonstrated that I/R injury of the heart can be attenuated by application of either LSTS or electroacupuncture (EA) at the PC 6 acupoint [32, 33]. To investigate the differences in myocardial protein expression between PC 6 stimulation by EA and by LSTS, animals were treated with either LSTS or EA stimulation at acupoint PC 6, and this was followed by harvesting of the heart at different time points for proteomic analysis. The results showed that either PC 6 stimulation by EA or PC 6 stimulation by LSTS had a cardioprotective effect against I/R injury. However, proteins related to energy production and inflammation, such as glycogen synthase kinase-3*α*, interleukin-1*β* converting enzyme (ICE), natural killer cell protease, and tumor necrosis factor receptors, were found to change in expression to a greater degree in response to EA treatment than to LSTS treatment. In contrast, LSTS increased to a greater extent than EA for various protective proteins, including creatine kinase and HSP70 [33].

**Figure 1:**
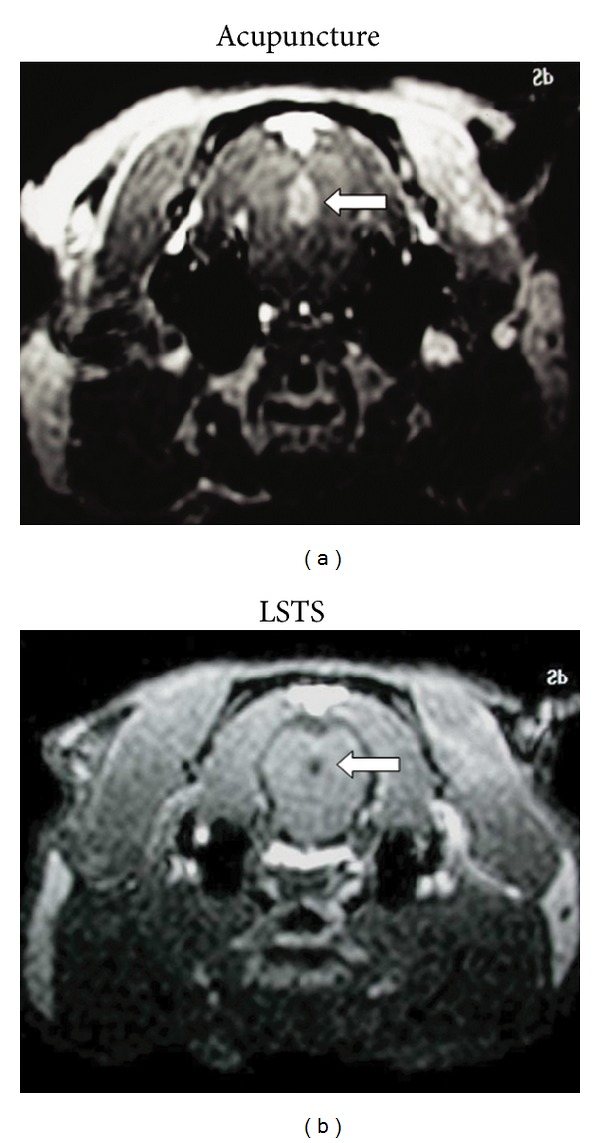
Different central manifestations between electrical acupuncture and local somatothermal stimulation at peripheral acupoints. Manganese-enhanced functional magnetic resonance imaging was performed in Sprague-Dawley rats after EA (a) at acupoint LI 4 and LSTS (b) at acupoint GB 24. The results showed that EA induced activation of pain-modulation nuclei such as the periaqueductal grey (PAG); however, in contrast, LSTS did not induce such activation.

There is consensus that acupuncture evokes complex somatosensory sensations and in this way may modulate the cognitive/affective perception of pain; this suggests that many effects are supported by the brain and various other central nervous system (CNS) networks. Modern neuroimaging techniques, such as functional magnetic resonance imaging (fMRI), have provided a means whereby brain activity in humans can be safely monitored. This type of approach is useful when mapping the neurophysiological correlates of acupuncture [54]. A meta-analysis of fMRI acupuncture studies has suggested that acupuncture is able to modulate activity within specific brain areas; however, more high quality studies with more transparent methodologies are needed to improve the consistency across the various different studies [55]. Nevertheless, using manganese-enhanced fMRI in animals, EA has been found to induce activation of pain-modulation nuclei such as the periaqueductal grey (PAG); however, in contrast, LSTS did not induce such activation (Figure 1). This supports the clinical observation that LSTS is not a modality for pain relief [56].

#### 3.1.7. ROS Plays an Important Role in LSTS-Induced Physiological Responses beneath the Acupoint

In order to elucidate the exact mechanism by which LSTS acts beneath the acupoint, LSTS was applied to the acupoint of animals, and the underling muscles were then collected at various time intervals after LSTS, namely, at baseline and at 5 min, 15 min, 30 min, and 60 min after baseline. The time-dependent profiles for free radical production and enzymatic scavenging activity were measured. The concentrations of reactive oxygen species, NO metabolites, and malondialdehyde were found to have increased significantly at 5 min after LSTS, whereas scavenging activity was reduced to its lowest level at 5 min (dismutase) and at 15 min (catalase and glutathione) after LSTS. Expression of HSP70 was significantly lower after LSTS when the animals were treated with an NO synthase inhibitor than in the control group without inhibitor. These results suggest that LSTS induces oxidative stress and a scavenging response in the underlying skeletal muscle and that this plays an initial role in the LSTS-induced Somatovisceral regulation mediated by the heat-sensory afferent loop [35].

#### 3.1.8. Limitations of LSTS

It should be noted that LSTS alone at the LR 14 and PC 6 acupoints induces an elevation in serum ALT/AST [31] and cardiac troponin T levels [33], respectively. In addition, LSTS alone on the calf muscle induces a mild elevation in serum creatine kinase MM isoform (CK-MM) levels [34], which supports the hypothesis that preconditioning by LSTS at peripheral acupoints acts as a stress and may cause cellular damage in the corresponding organs. Such sublethal damage is similar to that observed when ischemia preconditioning is carried out, and this seems to protect subsequent I/R injury [57, 58]. Minor injury may be able to initiate complex biochemical cascades that are able to protect against subsequent overwhelming I/R injury. The mechanisms of preconditioning are complicated. Various mediators, including nitric oxide (NO) and adenosine in the first few minutes to hours, inducible NO synthase (iNOS) and antioxidants enzymes in the first to fourth days, and *de novo* synthesized proteins such as HSP in the few days after preconditioning, have all been postulated to protect against I/R injury [59–62]. Thus, LSTS should be cautiously applied to or may be contraindicated for those patients with chronic liver or heart diseases.

### 3.2. The Nontemperature-Related Mechanisms of Action of Moxibustion

#### 3.2.1. The Effects of Herbs

When the traditional moxibustion technique is carried out, many herbs, including *Artemisia argyi* leaf and ginger, are widely used between the moxa sticks and the skin surface. Using gas chromatography-mass spectrometry (GC-MS) with solid-phase microextraction (SPME), a total of fifty-three compounds, including cylcofenchene, alpha-pinene, alpha-myrcene, D-limonene, caryophyllene, and germacrene D, were identified as well as two volatile components (borneol and borneol acetate) from *Artemisia argyi* flowers [63]. In addition, various nonvolatile substances, such as juniper camphor, caryophyllene oxide, and caryophyllene, have been found in a high proportion of moxa wools [64]. Furthermore, there is evidence supporting that the hypothesis that the increase in temperature induced by moxibustion increases the permeability of the skin to high molecular compounds [65], as well as acting as an aid to the entry of any topical application of salicylate [66].

#### 3.2.2. The Smoke Effects of Moxibustion

Previously, the anti-inflammatory effects of moxa smoke on NO production were demonstrated by Matsumoto H et al. using mouse macrophage-like Raw 264.7 cells. This study showed that the 50% inhibitory concentration (IC50) of lipopolysaccharide-induced NO production by moxa smoke (0.16%) was one order of magnitude lower than the 50% cytotoxic concentration (CC50) (4.67%). The inhibition of NO production by moxa smoke is probably due to both an inhibition of iNOS expression and an inhibition of radical scavenging activity [67]. Moreover, moxa smoke dose-dependently induces internucleosomal DNA fragmentation, activates caspases 3, 8, and 9, and modifies to some extent the expression of various apoptosis-related proteins (Bcl-2, Bad, and Bax) in HL-60 cells. These findings suggest that moxa smoke has potential as an antitumor agent [68]. It should be noted that there has been the advent of strict antismoking legislation in many countries, and as a result there are concerns about the potential effectiveness and toxicity of the volatiles produced by moxibustion. Up to now, no immediate concerns have been raised about the continued use of moxibustion as a therapeutic modality in traditional Chinese medicine [69].

#### 3.2.3. The Far Infrared (FIR) Effects of Moxibustion

It is reasonable to speculate that direct moxibustion with a traditional moxa stick may produce its therapeutic effects via thermal action, while traditional indirect moxibustion may act by producing both modest thermal activation and a sympathetic vibration at the skin surface [70]. Shen et al. demonstrated that intensity of infrared radiation produced by a traditional moxa stick was 43300.41 mV with a peak in the infrared spectrum at 3.5 *μ*m, while the respective radiation intensities of two control experiments using a smokeless moxa stick and a 555 cigarette were 31.15 mV and 37.03 mV with peaks of 7 *μ*m and 3.5 *μ*m, respectively. The infrared radiation intensities of the three traditional media used for indirect moxibustion, monkshood cake, ginger slices, and garlic slices were found to be 520.27 mV, 594.79 mV, and 681.87 mV, respectively, all with peaks around 7.5 *μ*m. These materials all produced similar spectra, which were quite different from those detected when slices of various control materials (cucumber and carrot) were used [70]. In addition, when moxibustion stimulation at the ST 25 acupoint was carried to treat animal ulcerative colitis, Wang et al. demonstrated that the infrared radiation intensity at fourteen wavelengths at the ST 25 acupoint were significantly different between the normal and model control groups. These findings suggest that biophysical mechanisms may be involved in the moxibustion treatment [71].

## 4. Adverse Events due to Moxibustion

Although traditional moxibustion has potential as a treatment, it is not entirely risk free, and several kinds of adverse events have been reported, including trauma [72], allergy, burns [73, 74], and infection. There is consensus that both the evidence-based efficacy of moxibustion and the level of patient safety of moxibustion need to be explored in more detail in order to clarify the usefulness and applicability of this ancient technique [75, 76].

## 5. Clinical Implication of LSTS

There is consensus that the expression of HSPs by prior sublethal hyperthermic preconditioning is able to attenuate the heat-induced cellular responses to a subsequent severe heat challenge [77]. The development of thermotolerance can be initiated by preconditioning animals, not only with repeated hyperthermia, but also with ischemia-reperfusion challenge or low doses of various chemical stressors [78–80]. In addition, accumulating evidence indicates that remote ischemia preconditioning attenuates I/R injury of the heart [81, 82]. In contrast to whole-body hyperthermia, which is likely to induce HSP expression in many organs, LSTS is a local heat stress and induces HSPs in specific corresponding visceral organs. LSTS at the PC 6 acupoint induces myocardial but not hepatic HSP70 expression, while LSTS at the LR 14 acupoint induces hepatic but not myocardial HSP70 expression. The fact that preconditioning LSTS at these two acupoints protect the heart and the liver against subsequent I/R injury, respectively, supports the concept that preconditioning LSTS at peripheral acupoints is a remote preconditioning technique.

## 6. Conclusion

Moxibustion is an ancient Chinese medical technique. The possible mechanisms by which moxibustion may work include temperature-related factors and nontemperature-related factors; the latter include smoke effects, herbal effects and biophysical effects (far infrared). Compared with whole-body hyperthermia or brief ischemia preconditioning, LSTS (an alternative to moxibustion that avoids skin damage) is an easily applicable preconditioning method for the prevention or treatment of overwhelming subsequent I/R injury. However, evidence-based studies of the efficacy of LSTS as well as safety studies are needed using large-scaled clinical trials in order that this ancient Chinese technique can pass muster with Western scientists.

## Figures and Tables

**Table 1:** Effects of LSTS on peripheral acupoints on visceral functions of the corresponding organs.

Acupoints	Visceral functions	Mechanisms		References
		Regulatory molecules	Serum
GB 24	Motility of SO ↓	NO ↑		Chiu et al., 1998 [28]

BL 40 and BL 36	Motility of anal sphincter ↓	NO ↑		Jiang et al., 1999 [29] Jiang et al., 2000 [30]

LR 14	Protects the liver from subsequent I/R injury	HSP70 ↑	I/R + LSTS versus I/R : ALT ↓ LSTS versus normal : ALT ↑ I/R + LSTS versus I/R : AST ↓ LSTS versus normal : AST ↑	Lin et al., 2001 [31]

PC 6	Protects the heart from subsequent I/R injury	HSP70 ↑	I/R + LSTS versus I/R : CPK ↓ I/R + LSTS versus I/R : CK-MB ↓	Chiu et al., 2003 [32] Tsou et al., 2004 [33]

BL 37	Protects the muscles from tourniquet-induced neuromuscular injury	ROS ↑ HSP70 ↑	I/R + LSTS versus I/R : CK-MM ↓ LSTS versus normal : CK-MM ↑	Pan et al., 2008 [34] Pan et al., 2012 [35]

SO: sphincter of Oddi; NO: nitric oxide; HSP70: heat shock protein 70; LSTS: local somatothermal stimulation; I/R: ischemia-reperfusion; ROS: reactive oxygen species; ALT: alanine aminotransferase; AST: aspartate aminotransferase; CPK: creatine phosphokinase; CK-MB: creatinine kinase-MB isoenzyme; CK-MM: creatine kinase-MM isoenzyme. Reference number is between square brackets.
